# Microflow Cytometers with Integrated Hydrodynamic Focusing

**DOI:** 10.3390/s130404674

**Published:** 2013-04-09

**Authors:** Marcin Frankowski, Janko Theisen, Andreas Kummrow, Peter Simon, Hülya Ragusch, Nicole Bock, Martin Schmidt, Jörg Neukammer

**Affiliations:** 1 Physikalisch-Technische Bundesanstalt (PTB), Abbestrasse 2-12, 10587 Berlin, Germany; E-Mails: andreas.kummrow@ptb.de (A.K.); peter.simon@ptb.de (P.S.); huelya@ragusch.de (H.R.); nicole.bock@ptb.de (N.B.); joerg.neukammer@ptb.de (J.N.); 2 Technische Universität Berlin, Strasse des 17. Juni 135, 10623 Berlin, Germany; E-Mails: janko.t@gmx.de (J.T.); schmidt@mfg.tu-berlin.de (M.S.)

**Keywords:** microfluidics, flow cytometry, lab-on-a-chip, single cell analysis, blood cell differentiation, hydrodynamic focusing, ultraprecision milling

## Abstract

This study demonstrates the suitability of microfluidic structures for high throughput blood cell analysis. The microfluidic chips exploit fully integrated hydrodynamic focusing based on two different concepts: Two-stage cascade focusing and spin focusing (vortex) principle. The sample—A suspension of micro particles or blood cells—is injected into a sheath fluid streaming at a substantially higher flow rate, which assures positioning of the particles in the center of the flow channel. Particle velocities of a few m/s are achieved as required for high throughput blood cell analysis. The stability of hydrodynamic particle positioning was evaluated by measuring the pulse heights distributions of fluorescence signals from calibration beads. Quantitative assessment based on coefficient of variation for the fluorescence intensity distributions resulted in a value of about 3% determined for the micro-device exploiting cascade hydrodynamic focusing. For the spin focusing approach similar values were achieved for sample flow rates being 1.5 times lower. Our results indicate that the performances of both variants of hydrodynamic focusing suit for blood cell differentiation and counting. The potential of the micro flow cytometer is demonstrated by detecting immunologically labeled CD3 positive and CD4 positive T-lymphocytes in blood.

## 1. Introduction

Mass production of microsystems has the potential to provide low-cost, disposable chips for complex cellular-based analyses including fundamentally new approaches. Microfluidic and nanofluidic systems have already found their place in modern analytics and seem to be one of the fastest growing branches in biotechnology and diagnostics [1–6]. Recently, the potential of microfluidics for single particle analysis was also spotted by the flow cytometry community [7]. Flow cytometry has a number of applications, e.g., in microbiology and marine biology, but it is mostly applied in laboratory medicine, in particular for differentiation and counting of blood cells [8]. This type of application was also demonstrated in a number of microchips [1,3,5,9–15]. Numerous commercial solutions show that such technology could establish the basis for simple and robust analytical systems being of particular relevance for point-of-care *in vitro* diagnostics with a wide range of applications in emergency medicine and intensive care. The main efforts made in this field, are focused to assure functionality which is comparable if not superior to conventional, large frame flow cytometric systems.

Particles are characterized by light scattering, fluorescence or electrical impedance in flow cytometry [8,16–20]. Fluid handling plays an important role for signal height and stability [14]. An essential part of the development of microflow cytometers is the control of the stream of micro particles in a flow channel [15]. Focusing of particles inside the flow channel significantly improves signal stability and prevents fouling of the channel walls. So far large frame flow cytometers use hydrodynamic focusing or occasionally acoustic focusing. Acoustic focusing is more popular for microfluidic chips since it does not require elaborate shaping of the fluidic channels [9,21,22]. Specifically designed fluidic channels perform focusing of the particle stream even without the use of sheath streams [23–25]. Sheathless focusing is advantageous for measuring dilute samples since these approaches concentrate the particles in space. Blood samples have very high cell concentrations that can reach above 10^6^ μL^−1^ (red blood cells) and usually require sample dilution to reach acceptable particle count rates avoiding extensive coincidence loss. Therefore, cells are more easily counted using hydrodynamic focusing, in which the sheath flow causes particles to pass the interaction region in single file. The sheath flow rate usually exceeds the sample flow rate by two to three orders of magnitude. Hydrodynamic focusing in microfluidic chips was for the first time implemented by Wolff and coworkers (“smoking chimney”) [26]. The sample stream is injected into a single sheath flow and the sample stream is hydrodynamically focused next in a tapered section. However, the fabrication of such a sample inlet is awkward. Other groups use different approaches to ensheath the sample stream from all sides [27–32]. Nevertheless, such solutions usually require precise adjustment of the flow rate ratios and are difficult to handle. On the other hand, recent studies demonstrate that even two-dimensional hydrodynamic focusing can be sufficient to measure cellular DNA content in tumor cells [33,34].

In this paper we compare two methods of hydrodynamic focusing that use a single inlet only for the sheath flow. One kind of microfluidic structures (cascaded focusing) exploits different channel heights for sample and sheath flows to envelope the sample stream in both lateral directions [10]. In the other case (spin focusing) the fluid flow is conducted in a fluid sheath flow with a swirling action that hydrodynamically focuses the flow in a (tapered) focusing channel section. The advantage of this novel approach is that the molding inserts used in chip production require lower aspect ratios.

The microfluidic chips discussed in this paper are designed for blood cell counting at high count rates without excessive coincidence losses [9,10,29,35–37]. We demonstrate that both structures presented here are suited for particle alignment in a micro flow cytometer for high throughput analysis of the blood cells. The potential of the chips for blood cell counting is demonstrated by the measurement of a titration series for fluorescently labeled lymphocytes.

## 2. Experimental Section

### 2.1. Design and Fabrication of the Microflow Cells

**Figure 1.**
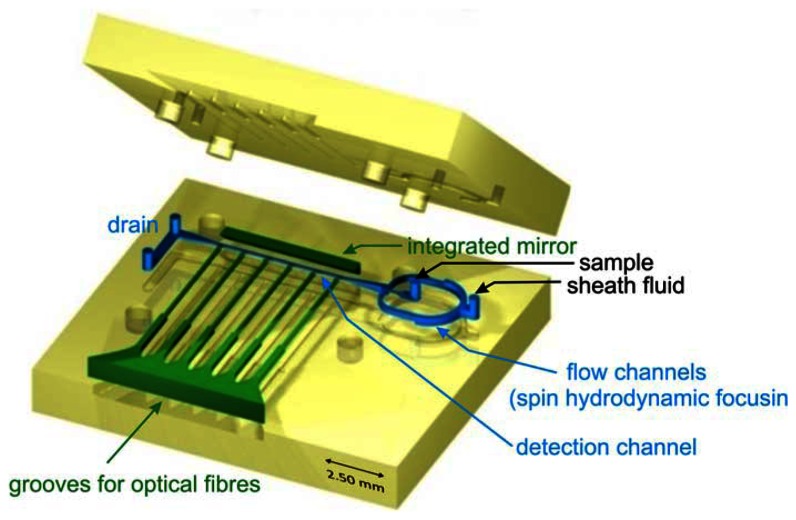
Layout of microfluidic structure featuring spin hydrodynamic focusing (the top and bottom parts prior to assembly). The hollow cavities of the microfluidic chip including flow channels (blue color), integrated mirror and six grooves to insert optical fibers (green) are “detached” from the body (yellow) to facilitate the overview on the layout. The detection channel (size: 100 μm × 100 mm) and the mirror are embossed in the bottom part of the structure. Hydrodynamic focusing channels and grooves for optical fibers are shared by both plates.

Microfluidic devices were fabricated using ultraprecision milling followed by hot embossing in transparent thermoplastic (polycarbonate). The structure of the chip is composed of top and bottom parts (size 20 mm × 20 mm) joined by laser welding (for details see [10]). The external fluidic connections were milled in the upper part and optionally, optical fibers were inserted prior to the joining of the structure. The layout of the microflow cytometer applying spin focusing configuration is shown in Figure 1. Combined milling and hot embossing techniques chosen here are superior to lithographic methods in obtaining fully 3-dimensional structures characterized by smooth transitions between different heights of the flow channels. Micromachining also allows the production of surfaces with optical quality and integration of optical components in the chip. A roughness of 5 nm for the reflecting plane at the angle of 45 degrees with respect to the joining plane of the structure was achieved using diamond tools. A mirror based on total internal reflection was integrated in the chip giving access for the observation of the flow channel in a plane perpendicular to the joining plane of the structure. Besides mirrors, integrated grooves allowed the insertion of optical fibers to guide a laser beam into the flow channel for excitation (mono-mode fiber) or detection of light scatter or axial light loss due to particles passing the interaction regions [10].

### 2.2. Hydrodynamic Focusing

Two different concepts of hydrodynamic focusing were implemented into the microflow cytometers for efficient and stable particle positioning in high throughput analyses of cells. All components required for hydrodynamic focusing are integrated into the microfluidic chips. The properties and corresponding layouts of microflow channels are summarized in Table 1. Both the two-stage cascade and the spin focusing require only one inlet for the sheath fluid besides the sample inlet and the outlet connection.

Ultraprecision milling allowed fabrication of mold inserts featuring smooth transition between different channel heights. Thus, the flow channel dimensions can be continuously reduced perpendicularly to flow direction and the joining plane. The values given in Table 1 show the difference between cascaded and spin focusing flow stages. The minimal curvature of channel walls was limited by the diameter of the end mill applied. In this case the curvature of joining points between the sample flow channel and sheath flow channel corresponds to 50 μm diameter for both focusing stages in the cascade design. The successive acceleration of cells in the cascaded focusing approach results in reduced shear forces which might destroy fragile cells during hydrodynamic focusing.

The sheath flow rate was adjusted by a pressurized reservoir and controlled by a flow meter (ASL 1430-24, Sensirion AG, Stäfa, Switzerland). Depending on the specific micro flow cytometer used the air pressure applied ranged from about 400 mbar to 800 mbar corresponding to sheath flow rates between 900 μL/min and 1,300 μL/min. Velocities varied from typically 0.5 m/s up to 3 m/s in the center of the flow channel. Ultrapure water was used as sheath fluid when analyzing fluorescent particles or rhodamine dye as sample stream. Isotonic solution (Isoton II, Beckman Coulter GmbH, Krefeld, Germany) was injected as sheath flow in blood cells measurements. The sample flow was controlled by a syringe infusion pump (model 540060, TSE Systems GmbH, Bad Homburg, Germany) using 1 mL syringes. The sample flow varied from 100 nL/min to about 300 μL/min.

The flow characteristics of the microchips were studied by fluorescence microscopy using aqueous solution of rhodamine 6G dye (concentration 30 μmol/L) excited with high pressure mercury arc lamp. The fluorescence of the hydrodynamically focused sample stream was imaged in both directions perpendicular to the direction of flow. Vertical profiles—Perpendicular to the assembly plane of the structure—were imaged *via* the on-chip integrated mirrors. Optimal image contrast was adjusted by varying the camera exposure time, typically in the range from 100 ms to 600 ms. Image analysis served to quantify the size of the sample stream as a function of the flow rate ratio for the sample and sheath flow (see [10] for details).

**Figure 2.**
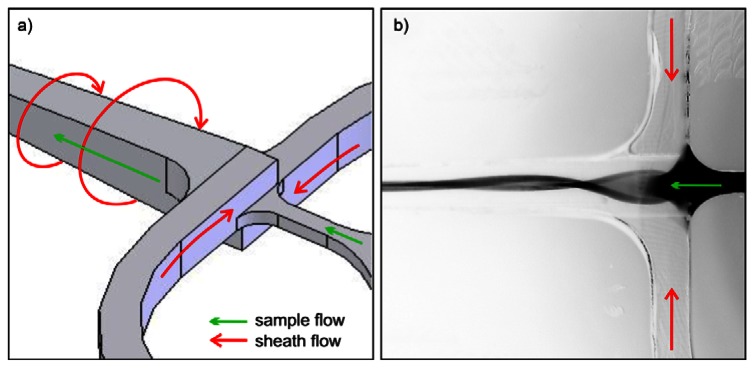
Principle of spin (vortex) hydrodynamic focusing integrated in a microfluidic chip. (**a**) Operation principle with sheath and sample flow directions. (**b**) Fluorescence image (inverted grey scale) of rhodamine dye 6G used as a sample fluid and excited with mercury arc lamp. To clearly demonstrate the principle of spin focusing the sample flow rate was set to 48 μL/min, above optimal conditions. Ultrapure water was used as sheath fluid with the total flow rate of 1,230 μL/min.

The sample stream is focused perpendicular to the joining plane by dividing the sheath flow in two symmetrical channels. At the sample injection outlet both flows are merged again. The structures shown in Table 1 use two different approaches to focus the sample stream also in vertical direction. The cascade focusing stages exploits significantly different heights of the flow channels for sample and sheath flow (1:8). Compared to single stage focusing, two stage focusing resulted in a considerably increased stability range with respect to the ratio of sample to sheath flow volume rates. To achieve stable conditions, the height of the channels in the cascade was chosen to be 1,000 μm compared to 125 μm flow channel dimension of the flow channel in the measurement region. On the other hand, in the spin focusing design the height of the fluidic channels is 210 μm (Table 1). The principle of operation is illustrated in Figure 2(a). Compared to other microfluidic structures, where up to four separate sheath flows (back, lifting and side sheath) are necessary [27,29,30] for full control of focusing and positioning of the sample stream only one inlet ensures efficient hydrodynamic focusing. The sample flow exhibits a spinning motion visualized by injecting a dye sample and observing its fluorescence image (Figure 2(b)).

### 2.3. Measurement and Data Acquisition

**Figure 3.**
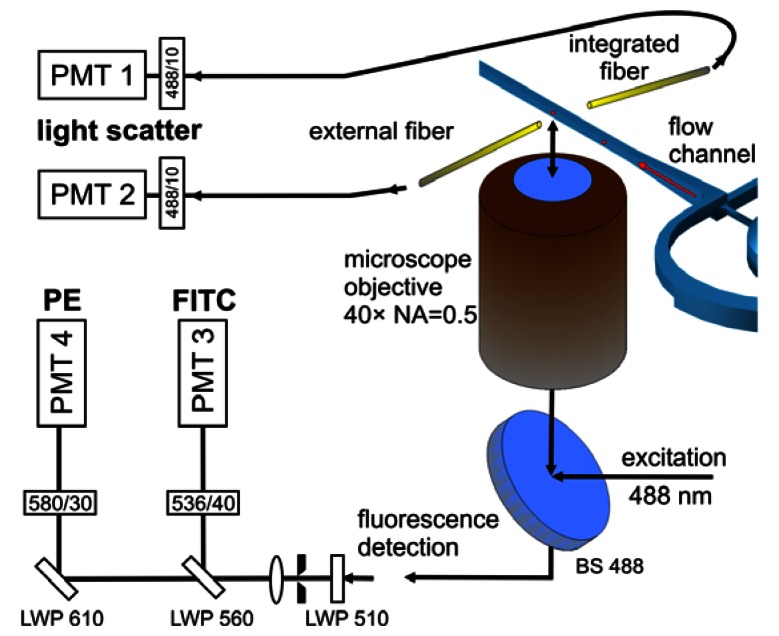
Schematic of the optical detection setup applied in measurements of microparticles and blood cells (not in scale). The microscope objective was used for laser excitation and fluorescence observation. The laser light scattered by either beads or blood cells were detected using integrated 105 μm (core diameter) optical fiber and/or external 600 μm optical fiber placed close to the flow channel. Wavelengths of maximum transmission of filters and corresponding bandwidths are given in nm (note: A bandpass filter of 535/35 was used in the measurements of the spin focusing chip).

Laser induced fluorescence from calibration microparticles and fluorescently tagged blood cells was detected by means of an inverted microscope equipped with a 40 × microscope objective (CDPlan 40 × ULWD, Olympus, Tokyo, Japan, numerical aperture NA = 0.5). The microfluidic chips were mounted on the motorized translation stages of an inverted microscope stand (Axiovert 200M, Carl Zeiss Microscopy, Göttingen, Germany). The sample was excited using the microscope objective in epi-illumination modality and a beam splitter for 488 nm. The microscope provides intermediate images at the exits of the side ports for signal detection or imaging camera. Figure 3 shows the arrangement applying fiber optics or epi-fluorescence path for measurements of fluorescence signals.

A solid state laser (Sapphire 488–200, Coherent, Santa Clara, CA, USA) was used as excitation source at a wavelength of (488 ± 2) nm in fluorescence measurement of calibration beads and immunologically stained blood cells. The output power of the laser was set in the range from 50 mW to 80 mW depending on the objectives of measurement. Obtaining stable light scatter or fluorescence signals from particles requires homogeneous illumination of the measurement zone. To this end the beam shaping optics was used to obtain an elliptically shaped laser spot in the detection channel, consisting of two pairs of spherical and cylindrical plano-convex lenses arranged in two Keppler configurations. The sizes of the elliptical foci were derived from fluorescence images and amounted to 7 μm by 87 μm (full width at half maximum, FWHM) for cascade focusing and to 16 μm by 38 μm for the spin structure. The orientations of the elliptical spots were chosen with the smaller axes being parallel to the flow direction. The extended size of the focus perpendicular to the flow direction significantly reduces the fluctuations of signal intensity caused by particles with trajectories being further away from the axis of the flow channel.

Laser light scattered by microparticles and blood cells was collected with multi-mode optical fibers mounted in the joining plane of the chip (internal fiber) or by an external fiber positioned outside the chip and aligned relative to the excitation spot (Figure 3). The numerical aperture of the integrated fiber is NA = 0.22 and the core diameter is 105 μm (AFS105/125Y, Thorlabs GmbH, Dachau, Germany). The externally applied fiber features a numerical aperture NA = 0.37 and a core diameter of 600 μm (Laser Components GmbH, Olching, Germany). The signal was detected directly at the exit end of the optical fibers by a photomultiplier (R3896, Hamamatsu Photonics K.K., Iwata-gun, Japan) equipped with an interference bandpass filter (center 488.0 nm, 10 nm bandwidth) mounted in front of the detector.

The laser induced fluorescence was selected from the intermediate microscopic image at the exit port of the microscope with a pinhole and was collimated by a lens. Fluorescence signals at different emission wavelengths from staining dyes (labeled cells or microparticles) were detected simultaneously with photomultipliers (Hamamatsu R3896 and R2949). Signals were spectrally separated using combinations of long pass and band pass filters.

Following logarithmic or linear amplification, signal pulse heights were detected, converted by analogue-to-digital converters, and binned in the appropriate number of histogram channels (CyFlow ML electronics; Partec GmbH, Muenster, Germany). In all measurements the light scatter signal served as trigger.

A CCD camera (AxioCam MR, 14 bit resolution, Carl Zeiss) mounted on optional exit port was used to monitor the flow channels and to image the sample flow to characterize hydrodynamic focusing. Real time imaging of the fluorescence of the dye flow was exploited to focus the microscope objective in the plane of the sample flow in advance to particle and blood cell investigations.

### 2.4. Sample Preparation

The quantification and calibration of fluorescence signals were performed taking suspensions of fluorescent particles, featuring eight different fluorescence intensities (Sphero™ Rainbow Calibration Particles RCP-30-5A, 8 peaks, 3.0–3.4 μm, Spherotech Inc., Lake Forest, IL, USA). The fluorescence intensity ranged from 100 to 330 000 MESF units (molecules of equivalent soluble fluorophore [38]). The peak intensities differed slightly between two batches of suspension applied in experiments. The typical concentration of particles diluted in ultrapure water amounted to 1,000 μL^−1^.

Blood samples were obtained from healthy volunteers who have given their informed consent in written form that the samples were used for research purposes. Samples of fresh venous blood were collected into collection tubes containing EDTA anticoagulant (ethylene diamine tetra-acetic acid). A blood aliquot (100 μL whole blood) was stained with fluorescently labeled antibodies targeting two subpopulations of T-lymphocytes. The staining solution contained two types of clusters of differentiation (antibodies), the CD3 and the CD4 conjugated with FITC (fluorescein isothiocyanate) and PE (phycoerythrin) fluorophores, respectively. The volume fraction of antibodies varied from 0.1 μL to 20 μL where the highest amount was recommended by the vendor (Simultest IMK-lymphocyte, BD Biosciences, San Jose, CA, USA) for routine measurements. After labeling, the stained blood aliquot was incubated for 15 minutes at room temperature. Subsequently, red blood cells were lysed and leukocytes were stabilized with a set of reagents (Cell Kit C05, Almedica AG, Giffers, Switzerland). The total volume of blood, lysis and stabilizing reagents amounted to 1,065 μL corresponding to approximately 9% blood solution which was used in measurements without further dilution.

## 3. Results and Discussion

### 3.1. Characterization of the Hydrodynamic Focusing

**Figure 4.**
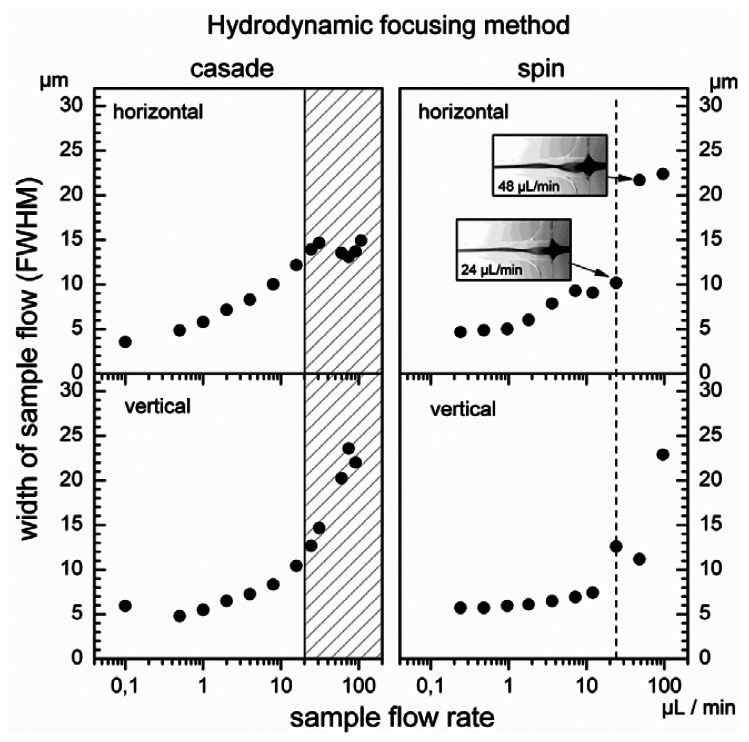
Widths of the sample flow measured as function of its volume flow rate at fixed total sheath flow rate. Horizontal width—measured parallel to the joining plane of the structure; vertical width—measured in plane perpendicular to the joining plane and the flow direction. The total flow rates were 1,000 μL/min for cascade and 1,230 μL/min for spin hydrodynamic focusing. Inserted fluorescence images (horizontal plane) illustrate the behavior of sample stream at flow rates of 24 μL/min and 48 μL/min.

In hydrodynamic focusing the particle size in the sample flow is in general of minor importance [20]. Thus, to define the operating range of microflow cytometer the sample flow was characterized with fluorescence microscopy using an aqueous solution of Rhodamine 6G. The size of the sample flow was directly determined from the FWHM of the intensity profiles measured in both directions with the CCD camera. The total volume flow rate was maintained at the constant level of 1,000 μL/min and 1,230 μL/min for cascade and spin focusing, respectively. The pressure applied to the sheath flow reservoir depends mainly on the cross section of the detection channel. Therefore, to maintain the same flow rate in both microfluidic chips, pressure was higher for the spin focusing chip, *i.e.*, 600 mbar *versus* 360 mbar for the cascade chip. The operating range for the ratios of sample to sheath volume flow rate was determined by varying the sample flow rate over three orders of magnitude. Results for both hydrodynamic focusing modalities are illustrated in Figure 4. In each case, the FWHM of the width of the sample stream was measured as function of the volume rates of the sample flow.

The minimum of the sample flow size (FWHM) determined for cascade as well as for spin focusing amounts to about 5 μm in both directions perpendicular to the flow vector. For the cascade arrangement, the half width in the structure assembly plane (horizontal) increases with an increase of the sample flow rate and achieves a maximum at a about 30 μL/min (Figure 4, left panel). Microscopic observation of the streaming fluids reveals that this behavior is correlated with an overflow of the second focusing stage by the sample fluid. In fact, there is a limit for stable focusing at 20 μL/min, which occurs already below that maximum. The corresponding sample half width amounts to 13 μm. Further enhancement of the sample flow rate leads to the observation of a relative minimum in the horizontal width at 80 μL/min while in vertical direction (perpendicular to the structure assembly plane) the sample flow continuously increases. The sample stream changes its geometry from an approximately circular cross section to an ellipse with the major axis oriented vertically.

Characteristics recorded for spin focusing in both lateral directions are shown on the right hand side of Figure 4. The width measured in horizontal and vertical direction do not follow the simple increase expected from the mass flow ratio of sample and sheath flow due to the spin focusing. The sample stream becomes elliptical and the orientation of the ellipse rotates with increasing sample flow rate (as illustrated by the fluorescence microscopic images). This causes the increased vertical and reduced horizontal width at the sample flow rate of 24 μL/min. At 48 μL/min the vortex makes an additional half rotation so that the horizontal width is particularly large and the vertical width is reduced.

The occurrence of the overflow defines rather well the stability limit for micro-device exploiting two-stage cascade focusing. Flow rates below this limit can be used as operating range for the microflow cytometer, *i.e.*, stable positioning of the particles for analysis are assured. The overflow regime for the cascade is indicated as hatched interval in Figure 4 (left). On the other hand, a sharp limit for focusing stability is not expected for the spin method. The rotation of the elliptical sample stream, however, compromises stable measurements above the flow sample rate of 24 μL/min (marked with the dashed line in Figure 4 on the right). This value can be regarded as the upper limit, below which spin structure provides stable particle positioning. The measurements indicate that the relative sample flow rates should be lower than 2% for both focusing methods. The results also show that for optimum performance, *i.e.*, resolution of small pulse heights, the relative flow rate should be limited to about 0.5%.

For both, cascade and spin focusing the experimentally determined widths exceed those expected from calculations. As discussed earlier, the mass conservation principle and FEM simulations predict a convergence of the sample flow size to zero for decreasing sample flow [10]. The differences are explained by typical exposure times of 300 ms used to derive the widths plotted in Figure 4. Faster fluctuations of the sample stream position below 300 ms cannot be measured and the experimental data correspond to an integrated width. However, for blood cell counting this measurement is representative, since measurements times are in the minute regime and the performance in particle counting is determined by the long term behavior of the microfluidic structures.

### 3.2. Characterization of Measurement Sensitivity and Resolution

**Figure 5.**
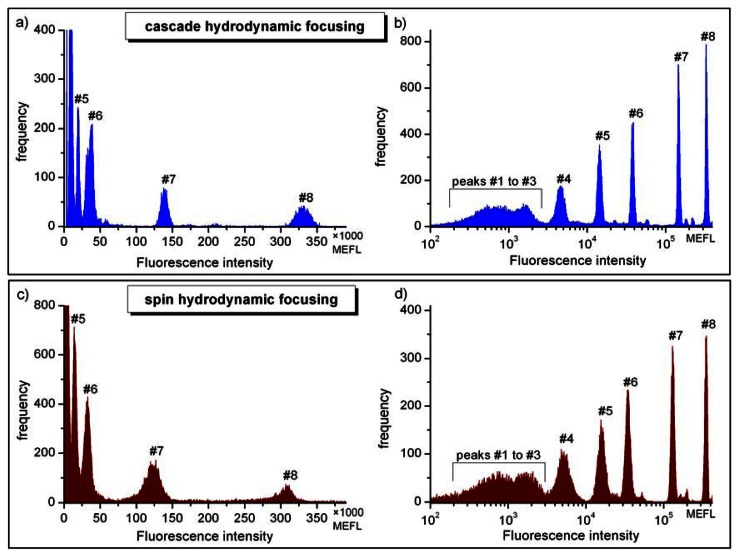
Pulse height distributions of fluorescence signals from calibration particles (Sphero™ Rainbow Calibration Particles). The sample contains eight particle populations corresponding to eight florescence intensity levels. Fluorescence was measured in the FITC detection channel. Top panel: histograms obtained with linear (**a**) and logarithmic (**b**) amplification for micro-device with the cascade 2-stage hydrodynamic focusing. Bottom panel: linear (**c**) and logarithmic (**d**) measurements with spin focusing. Fluorescence intensities are given in MEFL (molecules of equivalent fluorescein) units. Two batches of microparticle suspensions were used resulting in slightly different MEFL values in (a) and (b) compared to (c) and (d). Peaks were enumerated according to the calibration data sheets.

In optical flow cytometry the stability of the signal intensity is quantified by the pulse height distribution. To this end, the pulse height distributions of the fluorescence or light scatter signal are recorded for monodisperse calibration beads. The coefficient of variation (CV) of the pulse height distribution is indicative for the reproducibility and stability of measured signal amplitudes. The appropriate definition of fluorescence sensitivity for a flow cytometer was given by Chase and Hoffmann [39] as the ability to resolve subpopulations of dimly fluorescent particles, which in turn can be evaluated taking the respective CVs. This criterion was also applied to characterize the microflow cytometers described in this work. In this particular case, the assessment was based on the observation of laser induced fluorescence of the mixture of calibration beads exhibiting eight levels of the fluorescence intensity (Figure 5). The fluorescence was excited at a wavelength of 488 nm and detected at 536 nm through the optical bandpass filter of 40 nm bandwidth (Figure 2). The selected spectral range covers the fluorescence of blood cells stained with FITC. The fluorescence intensity is given in units of molecules of equivalent soluble fluorophore (MESF) [38] corresponding to the numbers of fluorescent molecules in solution. For the FITC fluorophore the applicable fluorescence intensity units are often denoted as MEFL (molecules of equivalent fluorescein) to indicate the detection channel used.

Since differentiation of blood cells based on immunofluorescence depends on the properties of the used staining reagents, the definition of the detection parameter range is related to particular assays and protocols. Requirements for sensitivity are obviously more demanding for cells which exhibit dim fluorescence upon antibody labeling. On the other hand, an ultimate detection limit is related to autofluorescence of cells and depends on the excitation source used. Therefore, we will focus the discussion on a particular objective, the differentiation of leukocytes in lysed peripheral blood sample presented in the next section.

Results obtained for the FITC fluorescence for both types of microchips are shown in Figure 5. Histograms were measured using linear (Figure 5(a,c)) or logarithmic amplification (Figure 5(b,d)) prior to pulse height analysis. Logarithmic amplification covering about 3.5 orders of magnitude allowed the observation of the entire fluorescence intensity range of calibration beads with the brightest peak at 330,000 MEFL and the (dim) group of not resolved peaks below 2,000 MEFL (Figure 5(b,d)). Although the mixture of calibration beads features eight fluorescence intensity levels, only the intensities of the seven brightest peaks above 500 MEFL (#2 to #8) are specified by the vendor for quantitative analysis. The intensity of the center of gravity of the non-fluorescent blank beads (peak #1) is not specified since autofluorescence sensitively depends on the filter characteristics of the flow cytometer used. Features observed in the vicinity of the main peaks at slightly higher fluorescence intensities and characterized by much lower abundance are due to particles agglomerates or signal coincidences. These events do not disturb analysis and the occurrence of coincidences can be reduced by further dilution of the sample. Non-resolvable features denoted as #1 to #3 were apparent for microchips using the cascade as well as spin focusing. It follows that in order to differentiate fluorescently tagged cells against unstained (negative) populations their fluorescence intensity should be at above 3,000 MEFL. Similar observations were made when detecting the fluorescence of PE labeled cells.

Comparison of the histograms in Figure 5(b,d) indicates noticeable differences in the widths of pulse height distributions recorded with both types of microfluidic structures. The reduced pulse height resolution of the histogram in Figure 5(d) histogram (spin focusing) is evident in the lower fluorescence intensity range around the peaks #4 and #5. The separation of the peak #4 distribution from events corresponding to dimmer particles is thus not as clear as in Figure 5(b). In the log histogram, higher channel numbers accumulate a wider range of signal data. Therefore we used linear amplification to quantify the observed differences in fluorescence distributions. The coefficients of variation (CV) values for the three brightest distributions (#7 and #8) seen in linear measurements (Figure 5(a,c)) were derived by fitting with Gaussian distributions. In this way values for the standard deviations and the peak centers were obtained. Typical CV values acquired for the brightest distribution (#8) were (3.0 ± 0.2)% for cascade focusing and (3.1 ± 0.2)% for the chip with integrated spin focusing. The corresponding sample flow rates amounted to 6 μL/min (cascade) and 4 μL/min (spin) and the particle count rates were 180 Hz and 45 Hz, respectively. It should be noted that for cascade micro-structures CVs smaller than 2%, *i.e.*, values only slightly larger than the value specified by the manufacturer of the calibration particles (1.64%) were reached by reducing the sample flow rate [12]. At lower fluorescence intensity for the bead population #7 the CV typically increased to (3.5 ± 0.2)% when measured using cascade focusing and up to (7.8 ± 0.2)% for spin focusing. Figure 5(b,d) reveal that the widths of the distributions, and in consequence the CVs, increase with the decrease of the fluorescence intensity. This tendency is stronger for spin focusing structure and to some extent correlates with higher fluorescence background, to be clearly noticed in Figure 5(c). On the other hand, the size of the excitation laser beam had to be adjusted to the narrower detection channel in the spin structure (100 μm × 100 μm *versus* 125 μm × 125 μm in cascade) and to minimize laser light scatter, since light scattering signals were acquired in this case with an external fiber (see Figure 3). Thus, the larger widths of the pulse height distributions measured with the spin focusing approach was predominately caused by the smaller focus size of 38 μm compared to 87 μm, which results in an increased sensitivity against mechanical fluctuations of the sample flow. This may explain why at present the cascade microflow cytometer is superior to its spin counterpart, in terms of resolving dimly stained beads. The implications for application in blood cell counting are discussed below.

### 3.3. Differentiation of Fluorescently Tagged Blood Cells

Differentiation of cells by optical flow cytometry typically involves detection of various measurement quantities. In large frame flow cytometers the light scatter is commonly detected at two different angles—forward scatter at about 10°, and side scatter at about 90° with respect to the excitation beam.

The number of fluorescence detection channels depends on the number of dye-conjugated antibodies applied to label the cells. Some protocols involve two or three types of antibodies to specifically stain subpopulation of blood cells, e.g., human peripheral blood lymphocytes.

**Figure 6.**
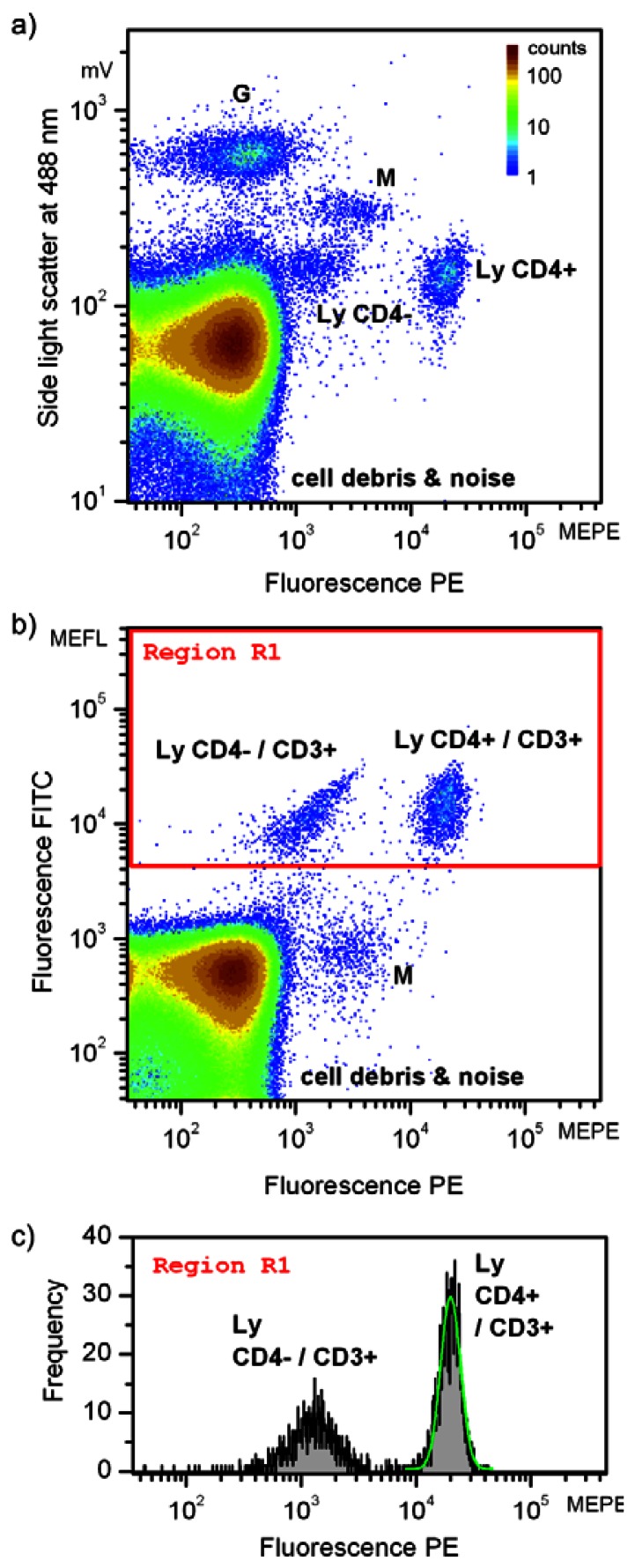
Three-parameter measurement of a blood sample labeled with anti-CD3-FITC and anti-CD4-PE antibodies specific for staining subpopulation of the T-lymphocytes (Ly). The number of events in the scatterplots (**a**) side light scatter *vs.* PE fluorescence and (**b**) FITC fluorescence *vs.* PE fluorescence are given in false colors. Subpopulations of leukocytes are labeled as G—granulocytes and M—monocytes. The histogram depicted in (**c**) represents events in “Region 1” of the scatterplot FITC fluorescence *vs.* PE fluorescence in (b). Intensity distributions of the subpopulation of CD3 positive and CD4 positive lymphocytes were fitted with lognormal distributions (marked with green contour) to derive the number of events (total number of events is 8390912 (a,b), and 2694 in region R1 (c)). Fluorescence intensities are given in MEFL (molecules of equivalent fluorescein) and MEPE (molecules of equivalent PE) units.

Double staining of human peripheral blood cells was applied using two antibodies conjugated with FITC, and PE stains, respectively, and targeting CD3 and CD4 antigens. While CD3 tags all T-lymphocytes, CD4 is used to identify CD4 positive (helper/inducer) T-lymphocytes. Laser excitation at 488 nm allowed the three-parameter measurement of blood suspension. Results are shown in Figure 6 using cascade focusing. Light scatter at the excitation wavelength was measured at orthogonally to the laser beam direction. Detection of white blood cells (leukocytes) with light scatter at 488 nm requires destruction of red blood cells (erythrocytes) by haemolysis. Otherwise, the signals arising from leukocytes would coincide with signals of erythrocytes, being more abundant by about three orders of magnitude.

In Figure 6(a,b), subpopulations of leukocytes including granulocytes (G), monocytes (M) and T-lymphocytes (Ly), cell debris (remains after cell haemolysis) and noise are identified. The false color scale indicates the frequency of occurrences and applies to both Figure 6(a,b). The total number of registered events amounted to about 840,000. Assignment of particular clusters of cell subpopulations is based on routine flow cytometric protocols [8]. Figure 6(a) depicts the intensities of the light scatter and fluorescence resulting from PE stain. Due to their intracellular granularity, granulocytes (G) can be clearly separated based on the orthogonal light scatter only. Because of their larger volume, also monocytes (M) exhibit higher light scatter intensities than lymphocytes (Ly). The target cell population, the T-lymphocytes are accessible combining light scatter signals and fluorescence intensities as demonstrated in Figure 6(a). Two different lymphocyte subpopulations are discernible, appearing as two clusters. The fluorescence intensity of both populations, the CD4 positive (CD4+) and the CD4 negative (CD4–) cells differ by about one order of magnitude. As common in flow cytometry, double staining (Figure 6(b)) was applied to unambiguously identify all lymphocytes. All T-lymphocytes were additionally tagged by CD3 antibodies and exhibit intensity above 4,000 MEFL units in the FITC fluorescence channel. It follows that besides the CD4–/CD3+ subpopulation of lymphocytes the target cells selected to demonstrate the performance of microflow cytometers are characterized being positively stained with both, CD3 and CD4 antibodies. Consequently, these cells are denoted as Ly CD4+/CD3+ in Figure 6(b). It should be noted that the weak fluorescence of CD4 negative cells detected in the PE fluorescence channel is due to spectral crosstalk from the FITC fluorescence. Furthermore, it is known that monocytes (M) might show a low expression of CD4 antigens and hence exhibit a PE fluorescence signal being slightly larger than CD4-lymphocytes in both scatterplots.

Changing volume concentration of the staining reagent in the blood aliquot is a typical approach to find optimal sample preparation conditions in cytometric analysis of fluorescently labeled cells. Such an analysis can also provide practical information on sensitivity, complementary to that performed with calibrated fluorescent particles. To this end we were decreasing the concentration of double CD3-FITC/CD4-PE staining reagent while observing the fluorescence intensity in PE channel. Figure 6(c) shows a histogram of the events delineated in Figure 6(b) by the rectangle denoted as “Region 1” (red frame). The number of events related to CD3 positive cells amounted to about 0.3% of all recorded signals. The subpopulation of CD3+/CD4+ lymphocytes was fitted with a lognormal distribution, the result is inserted in Figure 6(c) as a green contour.

**Figure 7.**
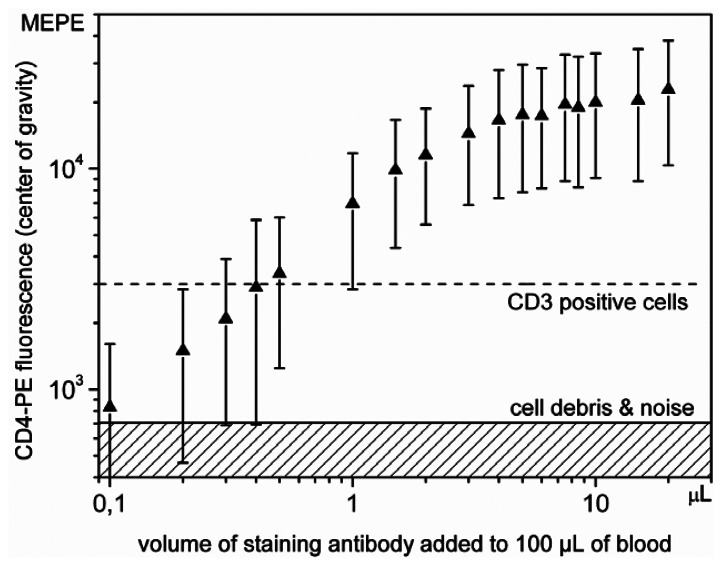
Dependence of the PE fluorescence intensity on the volume of the CD4-PE staining antibody solution added to 100 μL fresh blood sample. Quantitative titration was applied to demonstrate the detection sensitivity of the microflow cytometer. Fluorescence intensities are given in MEPE (molecules of equivalent PE) units.

Figure 7 shows the results of titration for CD4 antibodies by plotting the PE fluorescence intensity *versus* the volume of the staining reagent. The concentration of the reagent was changed from 0.1% up to the 20%, corresponding to 0.1 μL to 20 μL added to 100 μL blood. The widths of the CD3+/CD4+ distributions, covering 99.7% of these events, are given in molecules of equivalent PE (MEPE) units and plotted as bars in Figure 7.

The respective fluorescence intensities of the centers of gravity of the distributions are indicated as up triangles. The fluorescence intensity increases until it reaches a plateau for a concentration of about 5%. This concentration corresponds to the value recommended by vendors for blood samples featuring normal concentrations of lymphocytes. As expected, the relative widths of the CD4 clusters remarkably increase for low concentrations of the antibodies, since only few surface epitopes can be stained and the noise contribution increases. To derive minimum requirements for antibody staining, two PE fluorescence intensity levels were marked in Figure 7. The hatched area indicates background fluorescence of cell debris and noise as denoted in Figure 6(a and b). The solid horizontal line close to 700 MEPE units indicates the lower intensity limit needed to identify the CD4 positive cells. Clear separation of the CD4 positive and CD4 negative cells was achieved at concentrations above 0.5%, for which their distributions do not overlap anymore. The determined detection limit, *i.e.*, a relative volume of 0.5% antibody staining solution, refers to the specific staining protocol applied. The double staining procedure and the interference originating from spectral crosstalk of the CD3-FITC labeled T-lymphocytes result in the background fluorescence in the PE detection channel. Hence, the background fluorescence indicated by the dashed line in Figure 7, representing the separation of CD4– and CD4+ cells, is designated as CD3 positive because it also characterizes the maximum crosstalk fluorescence of CD3+/CD4– cells. On the other hand, double staining is necessary to ensure identification of all T-lymphocytes. It follows that the detection sensitivity of the microfluidic structure is determined by fluorescence of not targeted cells rather than by fluorescent background not caused by particles, as usual in flow cytometric analysis [39].

Evaluation of novel microfluidic solutions is usually based on comparative measurements performed with conventional, large frame flow cytometers. An extensive discussion for the cascade microflow cytometer is provided in our earlier work [12] where the blood samples were simultaneously analyzed using the high-end cell sorter (Mo-Flo, Beckman-Coulter). It was concluded that in spite of smaller sensitivity compared to Mo-Flo instrument, the microflow cytometer demonstrates equivalent performance in differentiation of blood cells exhibiting moderate fluorescence intensity.

## 4. Conclusions and Outlook

We have demonstrated that optical microflow cytometers based on two different concepts of integrated hydrodynamic focusing can be exploited for particle and blood cell analyses. On-chip implementation of three dimensional microfluidic structures in thermoplastic materials was accomplished by ultraprecision milling followed by hot embossing. Unlike the one-step lithography approach, the technique applied here allowed to achieve smooth transitions between flow channel widths in both lateral directions perpendicular to the flow vector. Effective hydrodynamic positioning of particles within 5 μm in the center of the detection channel was realized. The focusing method based on a flow cascade exploited channels of different heights to ensheath the sample stream in two subsequent stages. Alternatively, we applied a spinning sheath flow which focused the sample stream conducted into the tapered channel. In both structures the number of fluidic ports was reduced to minimum resulting in simple handling of the fluids and operation of the microflow cytometers in a wide range of flow rates and particle velocities from 0.5 m/s to 3 m/s. Besides the lower level of design complexity, the advantage of spin focusing structures is that smaller aspect ratios for mold inserts are sufficient and correspondingly chip production becomes easier.

The microfluidic chips discussed in this paper were designed for high throughput optical analysis of fluorescently labeled blood cells. Applied micromachining and assembling techniques allowed integration of optical elements including on-chip mirrors. Mirrors were used to quantify the size of the focused sample stream in two lateral directions and to determine the parameters for stable flow.

We have evaluated the measurement sensitivity of microflow cytometers using suspensions of calibration particles exhibiting eight levels of fluorescence intensities. The coefficients of variation (CV) determined for pulse height distributions of fluorescence signals were used as indicators of the stability and reproducibility of measurements. The sensitivity was evaluated in a spectral range corresponding to the fluorescence emission of FITC dye used in routine staining of blood cells. CV obtained for the brightest beads (about 3 × 10^5^ MEFL) was about 3.0% for the cascade focusing. Corresponding value for the spin focusing structure was about 3.1% for 1.5 times lower sample flow rate. The analysis of the pulse height distributions of dimly fluorescing calibration beads revealed that the cascade microchip performed better than the spin focusing microchip. In view of the same laser source, detectors and signal processing electronics used to test both microflow cytometers, we attributed the increase of the CVs observed for the spin focusing structure to the smaller focus size of the excitation laser beam.

Although CVs down to 1% can be achieved in modern commercially available benchtop flow cytometers, the typically specified values are between 2% and 5%. Apart from high resolution studies of cellular DNA content [40], such pulse height resolutions are sufficient for light scatter and immunofluorescence based cellular analyses due to significantly larger biological variations of the signal distributions measured.

To point out the potential of the microflow cytometers in differentiation of the fluorescently tagged blood cells we performed test measurements on peripheral blood T-lymphocytes. Measurement of the CD4 and CD3 labeled lymphocytes is used in medicine to quantify progression of the HIV disease by determining concentration of the CD4 positive cells in blood of infected patients [41]. As we demonstrated here, the microflow cytometer exploiting integrated cascade hydrodynamic focusing is fully capable to perform such a task. Applying light scatter and double immunostaining to erythrocyte depleted blood sample we were able to differentiate monocytes, granulocytes and the subpopulations of lymphocytes. In addition to fluorescent beads, sensitivity of the micro-device was verified by the CD4-PE stain titration showing that a significant decrease in fluorophore concentration does not compromise efficient differentiation of cells by this micro-device. At present the microflow cytometer exploiting spin focusing has a somewhat lower sensitivity than the cascade chip. However, it is still applicable for the detection of CD4 positive cells and for a number of biomedical applications which do not require sensitive fluorescence detection, such as cell viability testing using strongly fluorescing calcein or ethidium-homodimer-1 staining [42].

Improvement of the optical quality and reducing background fluorescence of the materials used in microfabrication is a priority in achieving higher sensitivity and resolution of micro-devices. Cyclic olefin copolymers for example are already known as a promising alternative to polycarbonate used in this work. On-chip integration of collection optics with high numerical aperture and laser beam shaping elements could also be an asset. Further development would focus on the integration of the sample preparation to proceed to the application of single used microfluidic chips. High detection sensitivity of the analysis unit combined with micro-compartments for automated cell labeling would not only reduce the costs of assays decreasing with the amount of reagents used, but also allow for precise determination of the volume concentration.

## Figures and Tables

**Table 1.** The 2-dimensional hydrodynamic focusing concepts applied in microflow cytometers.

**Design/Features**	**Cascade**		**Spin (Vortex)**	
**Layout**	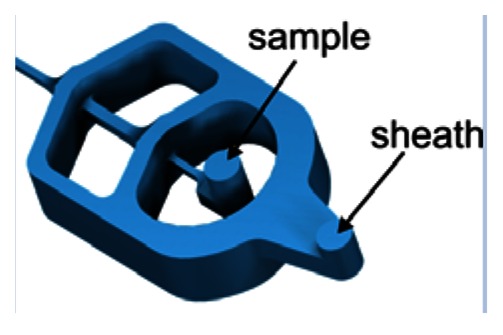		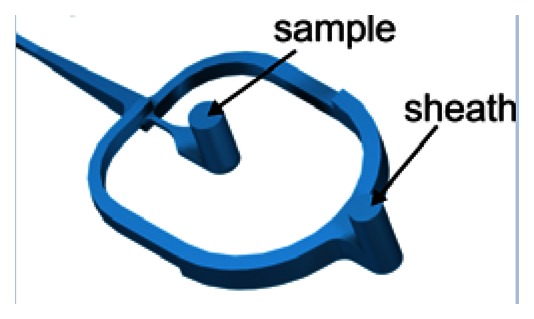	
**Sheath flow channel**				
width	1st stage: 300 μm;		125 μm	
height	2nd stage: 270 μm		210 μm	
	1,000 μm			
**Sample flow channel**	1st stage: 125 μm × 125 μm		width: 70 μm	
	2nd stage: 200 μm × 200 μm		height: 70 μm	
**Detection channel**				
size	125 μm × 125 μm		100 μm × 100 μm	
length	5.7 mm		3.4 mm	
